# From Africa to Antarctica: Exploring the Metabolism of Fish Heart Mitochondria Across a Wide Thermal Range

**DOI:** 10.3389/fphys.2019.01220

**Published:** 2019-10-04

**Authors:** Florence Hunter-Manseau, Véronique Desrosiers, Nathalie R. Le François, France Dufresne, H. William Detrich, Christian Nozais, Pierre U. Blier

**Affiliations:** ^1^Département de Biologie, Université du Québec à Rimouski, Rimouski, QC, Canada; ^2^Biodôme de Montréal, Montréal, QC, Canada; ^3^Department of Marine and Environmental Sciences, Northeastern University Marine Science Center, Nahant, MA, United States

**Keywords:** temperature, adaptation, pyruvate dehydrogenase complex, carnitine palmitoyl transferase, hydroxyacyl-CoA dehydrogenase, electron transport system, energy metabolism, fatty acid metabolism

## Abstract

The thermal sensitivity of ectotherms is largely dictated by the impact of temperature on cellular bioenergetics, particularly on mitochondrial functions. As the thermal sensitivity of bioenergetic pathways depends on the structural and kinetic properties of its component enzymes, optimization of their *collective* function to different thermal niches is expected to have occurred through selection. In the present study, we sought to characterize mitochondrial phenotypic adjustments to thermal niches in eight ray-finned fish species occupying a wide range of thermal habitats by comparing the activities of key mitochondrial enzymes in their hearts. We measured the activity of four enzymes that control substrate entrance into the tricarboxylic acid (TCA) cycle: pyruvate kinase (PK), pyruvate dehydrogenase complex (PDHc), carnitine palmitoyltransferase (CPT), and hydroxyacyl-CoA dehydrogenase (HOAD). We also assayed enzymes of the electron transport system (ETS): complexes I, II, I + III, and IV. Enzymes were assayed at five temperatures (5, 10, 15, 20, and 25°C). Our results showed that the activity of CPT, a gatekeeper of the fatty acid pathway, was higher in the cold-water fish than in the warmer-adapted fish relative to the ETS (complexes I and III) when measured close to the species optimal temperatures. The activity of HOAD showed a similar pattern relative to CI + III and thermal environment. By contrast, PDHc and PK did not show the similar patterns with respect to CI + III and temperature. Cold-adapted species had high CIV activities compared to those of upstream complexes (I, II, I + III) whereas the converse was true for warm-adapted species. Our findings reveal a significant variability of heart mitochondrial organization among species that can be linked to temperature adaptation. Cold-adapted fish do not appear to compensate for PDHc activity but likely adjust fatty acids oxidation through higher activities of CPT and HOAD relative to complexes I + III.

## Introduction

Mitochondrial ATP production depends on the interactions of various metabolic pathways. These pathways contain a variety of enzymes which are differentially affected by temperature. Thus any changes in temperature will induce a shift in the relative control strength of key steps of aerobic pathways (Blier et al., 2014). Lasting changes in environmental temperature will subject mitochondrial energy production to selective pressure to compensate for the adverse thermal impacts on the limiting or controlling steps of respiration.

In fish, the cardiovascular system is strongly affected by temperature and part of this sensitivity appears to be dictated by mitochondrial functions (Iftikar and Hickey, 2013). The thermal sensitivity of state 3 mitochondrial respiration in the heart of the Atlantic wolffish (*Anarhichas lupus*) has been shown to be similar to that of the pyruvate dehydrogenase complex (PDHc) but to differ from the sensitivity of other mitochondrial enzymes (Lemieux et al., 2010a, b). This has led authors to suggest that state 3 may be limited at low temperature in part by the activity of PDHc when mitochondria are fed with pyruvate. Moreover, at low temperature, PDHc activity was much higher in the heart mitochondria of wolffish, a cold-temperate adapted ectotherm than in the rat, consistent with a compensatory adjustment of this enzyme (Lemieux et al., 2010a, b). Further work on *Drosophila simulans* revealed an excess of complex IV (CIV) capacity at low temperature, indicating upstream limitation in the electron transport system (ETS) with PDHc as the likely cause of this limitation (Pichaud et al., 2010, 2011). These authors further noted that *Drosophila* CIV controlled mitochondrial metabolism at high temperature but not at low temperature. Takeuchi et al. (2009) showed that *D. simulans* mutants with a deficiency causing elevation of intracellular calcium concentration also had higher PDHc activity in colder environments compared to congeners without the mutation. Since calcium is a PDHc activator, the authors proposed that induction of this enzyme led to tolerance of colder temperatures by the mutants. Together, these results support the hypothesis that an excess of CIV activity at low temperature occurs due to a rate-limiting step upstream in ectotherms (Blier et al., 2014).

Another significant pathway for fish bioenergetics is the lipid metabolism. Lipids are important catabolic substrates for fish heart as they have the greatest energy density of the different fuels of metabolism, which makes them perfectly suited for energy-expensive activities (Magnoni and Weber, 2007; Weber, 2011). Circulating triacylglycerol (TAG) levels in resting rainbow trout (*Oncorhynchus mykiss*) have been shown to be sufficient to sustain exercise, which probably explains why TAG levels do not increase with long-term activity (Magnoni et al., 2008). A predominance of lipid oxidation over protein and carbohydrate in rainbow trout swimming at different speeds (Lauff and Wood, 1996) further portrays the importance of lipid oxidation in fish energetics.

Despite the importance of lipid metabolism, relatively few studies have evaluated the impact of temperature on mitochondrial fatty acid oxidation. Fatty acid molecules require a transporter, carnitine palmitoyltransferase (CPT), to translocate them through the mitochondrial membrane. CPT is embedded in the mitochondrial membrane, which tends to become more rigid as temperature declines (Weber, 2011) and therefore impair lipid transport. Conversely, elevation of the activities of enzymes of the β-oxidation pathway [in particular β-hydroxyacyl-CoA dehydrogenase (HOAD)], by enhancing the concentration gradient of lipids across the inner membrane (Weber, 2011), may offset reduced CPT activity at low temperature. In a comparison of mitochondrial metabolism between two ecotypically similar fishes, one Antarctic (*Trematomus newnesi*) and one temperate (*Tautoga onitis*), Crockett and Sidell (1990) found significantly higher activities of CPT (1.9-fold increase) and HOAD (27.2-fold) in the polar species. Two demersal fish species, the Antarctic *Gobionotothen gibberifrons* and the temperate *Myoxocephalus octodecimspinosus*, gave similar, but less pronounced, trends: the polar fish showed a 1.3-fold higher activity of CPT and a 6.8-fold higher activity of HOAD. Based on these results, Crockett and Sidell (1990) suggested that fatty acids are an important fuel source for metabolism in polar fish.

Determination of the temperature dependence of mitochondrial function and the activities of key enzymes should help us identify components that are able to acclimatize or adapt to thermal variation. Based on previous studies (summarized in Blier et al., 2014), we hypothesize that the activities of enzymes responsible for providing substrates to the tricarboxylic acid (TCA) cycle (PDHc, CPT, and HOAD), have been increased relative to those of ETS enzymes (complexes I, II, III, and IV), in cold-living species compared to temperate or warm adapted species. If this hypothesis is correct, then our results will provide a new framework for monitoring evolutionary compensation of mitochondrial metabolism in fish taxa across a wide range of thermal habitats. Any divergence in the relative proportion of key enzymes activities, when measured at or close to optimal temperature, will show the evolutionary plasticity of heart mitochondrial phenotypes and point out characters that could be under selective pressure to adapt to changing environmental conditions.

## Materials and Methods

### Experimental Animals

Eight species with different thermal requirements were used to conduct the experiment. Two cold-water notothenioid species, the marbled notothen (*Notothenia rossii*, Richardson, 1844; *T*_opt_ = −1.9 to 2°C, Strobel et al., 2012) and the humphead notothen (*Gobionotothen gibberifrons*, Lönnberg, 1905; T_opt_ = −1.9 to 2°C, Bilyk and DeVries, 2011), were collected by bottom trawling from the ARSV *Laurence M. Gould* near Palmer Station, Antarctica. Two North Atlantic cold-temperate species acclimated to 10°C, the spotted wolfish (*Anarhichas minor*, Olafsen, 1772; *T*_opt_ = 10.3°C, Hansen and Falk-Petersen, 2002) and the Atlantic cod (*Gadus morhua*, Linnaeus, 1758; *T*_opt_ = 9 to 12°C, Pederson and Jobling, 1989), were provided by the Maurice Lamontagne Institute (Department of Fisheries and Oceans of Canada, Mont-Joli, QC, Canada). Two temperate water species acclimated to either 13 or 15°C, the Arctic char (*Salvelinus alpinus*, Linnaeus, 1758, *T*_opt_ = 12 to 15°C, Jobling et al., 1992) and the striped bass (*Morone saxatilis*, Walbaum, 1792, *T*_opt_ = 18°C, Cox and Coutant, 1981) were also used. Arctic char were provided by a fish farm (Pisciculture des Monts de Bellechasse Inc., Saint-Damien-de-Buckland, QC, Canada) and bass by the Ministère des Forêts, de la Faune et des Parcs du Québec (Fish hatchery of Baldwin-Coaticook, QC, Canada). Two warm-water species, acclimated to 23–25°C, Nile tilapia (*Oreochromis niloticus*, Linnaeus, 1758; *T*_opt_ = 30.1°C, Xie et al., 2011) and common carp (*Cyprinus carpio*, Linnaeus, 1758; *T*_opt_ = 25°C, Watanabe et al., 1996), were provided by the Biodôme of Montreal (Montreal, QC, Canada). Some Nile tilapia were supplied by Urban Food Ecosystems (Montreal, QC, Canada). All manipulations were performed in agreement with ethical regulation of animals use for research in Canada [permit #CPA-65-16-174 from the animal ethics committee of the Université du Québec à Rimouski (Rimouski, QC, Canada)].

Four of the species belong to the more basal orders of the clupeocephala taxa (*G. morhua*, Gadiformes; *S. alpinus*, Salmoniformes; *C. carpio*, Cypriniformes, and *O. niloticus*, Cichliformes) which cover a range of optimal temperatures from 9 to 30°C. The four other species belong to the more derived order of Perciformes and cover a range of optimal temperatures from approximately 0–18°C. In the group of non-perciformes, the species more closely related to perciformes is *O. niloticus* with the highest optimal temperature of 30°C. We chose species in these two groups to ensure overlap in optimal temperature range that would be independent of phylogeny. Furthermore, there is no clear association of optimal temperature with phylogenic relationship. For example, *O. niloticus* (*T*_opt_ = 30.1°C) is more distant from *A minor* (*T*_opt_ = 10.3°C) than from both notothenoid species (*T*_opt_ close to 0°C) and the most distant species from *O. niloticus* is *C carpio*, with the most similar optimal temperature (25°C) (see the phylogeny of ray-finned fishes by Hughes et al., 2018). This allowed to avoid bias in the relation of metabolic characters with optimal temperature that would be associated to phylogenetic inertia.

### Tissue/Sample Preparation

After recording mass and length (total and standard), fish were euthanized with tricaine methanesulfonate (MS-222 0.25 g/L). Hearts were dissected, weighed, flash frozen (in liquid nitrogen) and stored at −80°C until analyses. For each species and analysis, an average of five hearts were pooled to provide sufficient material. The pools were made randomly to amount to comparable weight between the pool of the same species. The majority of the pools contain mature and immature fish. Using this method, three pools (*n* = 3) were made for *N. rossii*, *G. Morhua*, *O. niloticus*, and *C. carpio*, four pools (*n* = 4) were made for *S. Alpinus*, *G. gibberifrons*, and *A. minor* and five pools (*n* = 5) were made for *M. saxatilis*. Hearts were thawed on ice in a cold room (+4°C) and then homogenized on ice in 50 mM potassium phosphate buffer pH 8.0 (with 1 mM EDTA) using a Polytron (PT 2500E, Kinematica AG, Bohemia, NY, United States) for three periods of 10-s each. Homogenates were flash frozen, then stored at −80°C until use.

### Enzymatic Assays

The enzymatic activities were determined at 5, 10, 15, 20, and 25°C for four species (wolfish, cod and the two notothens); at 10, 15, 20, and 25°C for tilapia, Arctic char and bass; and at only 15 and 25°C for the carp (due to limited quantity of tissue).

Assays at 25°C were performed with a UV/Vis spectrophotometer (Ultrospec 3100pro UV/visible spectrophotometer, GE Healthcare, Mississauga, ON, Canada) equipped with a temperature-controlled cuvette holder connected to a 25°C water circuit or with a microplate reader with temperature control (EnVision Multilabel Plate Reader 2104-0010A, PerkinElmer, Waltham, MA, United States). Activities at 15 and 20°C were performed with a second microplate reader (Power Wave XS2, BioTek, Winooski, VT, United States), whereas activities at 5 and 10°C were measured in a cold room with a UV/Vis spectrophotometer (Ultrospec 2100pro UV/visible spectrophotometer, GE Healthcare, Mississauga, ON, Canada).

Protocols used for enzymatic assays were adapted as follows: PK from Pelletier et al. (1994); CII from Lemieux et al. (2010a); ETS from Madon et al. (1998); CI from Janssen et al. (2007); and PDHc, CPT, HOAD, CS, and CIV from Thibault et al. (1997). All measurements were obtained at least in duplicates.

Pyruvate kinase (PK, EC 2.7.1.40) activity was measured for four min at 340 nm to follow the disappearance of NADH (extinction coefficient ε_340_ = 6.22 ml cm^–1^ μmol^–1^) in a reaction medium containing 50 mM imidazole-HCl, 10 mM MgCl_2_, 100 mM KCl, 5 mM ADP, 0.15 mM NADH, 5 mM phosphoenolpyruvate and 0.6 U ml^–1^ lactate dehydrogenase, pH 7.4.

Pyruvate dehydrogenase complex (EC 1.2.4.1) activity was measured at 500 nm to follow the reduction of *p*-iodonitrotetrazolium violet (INT, extinction coefficient ε_500_ = 15.4 ml cm^–1^ μmol^–1^) in a reaction medium containing 50 mM Tris–HCl, 0.05% (v/v) Tween 20, 1 mM MgCl_2_, 2.5 mM NAD, 0.5 mM EDTA, 0.1 mM coenzyme A, 0.3 mM oxalate, 0.6 mM INT, 6 U ml^–1^ lipoamide dehydrogenase, 0.2 mM thiamine pyrophosphate and 5 mM pyruvate (omitted for the control), pH 8.0. Samples were incubated on ice with 80 mM MgCl_2_, centrifuged at 10,000 *g* (4°C) and the pellet was discarded prior to the analysis.

Carnitine palmitoyltransferase (EC 2.3.1.2) activity was measured at 412 nm to follow the reduction of 5,5′-dithiobis-2-nitrobenzoic acid (DTNB, extinction coefficient ε_412_ = 14.15 ml cm^–1^ μmol^–1^) in a reaction medium containing 75 mM Tris–HCl, 1.5 mM EDTA, 0.25 mM DTNB, 0.035 mM palmitoyl-CoA and 2 mM L-carnitine (omitted for the control), pH 8.0. Samples were centrifuged at 3000 *g* (4°C) and the pellet was discarded prior to the analysis.

β-hydroxyacyl dehydrogenase (HOAD, EC 1.1.1.35) activity was measured at 340 nm to follow the disappearance of NADH (extinction coefficient ε_340_ = 6.22 ml cm^–1^ μmol^–1^) in a reaction medium containing 100 mM triethanolamine-HC1, 5 mM EDTA, 1 mM KCN, 0.115 mM NADH, and 0.05 mM acetoacetyl-CoA (omitted for the control), pH 7.0. Samples were centrifuged at 3000 *g* (4°C) and the pellet was discarded prior to the analysis.

Citrate synthase (CS, EC 2.3.3.1) activity was measured at 412 nm to follow the reduction of 5,5′-dithiobis-2-nitrobenzoic acid (DTNB, extinction coefficient ε_412_ = 14.15 ml cm^–1^ μmol^–1^) using a reaction medium containing 100 mM imidazole-HCl, 0.1 mM DTNB, 0.1 mM acetyl-CoA and 0.15 mM oxaloacetic acid (omitted for the control), pH 8.0.

NADH:ubiquinone reductase (CI, EC 1.6.5.3) activity was measured at 600 nm to follow the reduction of 2,6-dichloroindophenol (DCIP, extinction coefficient ε_600_ = 19.1 ml cm^–1^ μmol^–1^). Homogenates were centrifuged at 3000 *g* (4°C) prior to the analysis. The supernatants were incubated for 10 min at the proper assay temperature in a reaction medium containing 100 mM imidazole, 2.5 mg ml^–1^ BSA, 5 mM MgCl_2_, 4 μM antimycin A, 10 mM sodium azide, 50 μM DCIP, 65 μM ubiquinone (coenzyme Q1) and 5 μM rotenone (only for the control), pH 8.0. The reaction was initiated by addition of 0.14 mM NADH.

Succinate dehydrogenase (CII, EC 1.3.5.1) activity was measured at 600 nm to follow the reduction of 2,6-dichloroindophenol (DCIP, extinction coefficient ε_600_ = 19.1 ml cm^–1^ μmol^–1^). Homogenates were incubated for 5 min at the desired assay temperature in a reaction medium containing 100 mM imidazole, 20 mM succinate, 4 μM antimycin A, 5 μM rotenone and 10 mM sodium azide, pH 7.2. The reaction was started by addition of 50 μM DCIP and 65 μM ubiquinone (omitted for the control).

CI + CIII activity was measured at 490 nm to follow the reduction of *p*-iodonitrotetrazolium violet (INT, extinction coefficient ε_490_ = 15.91 ml cm^–1^ μmol^–1^) in a reaction medium containing 100 mM potassium phosphate, 0.85 mM NADH, 2 mM INT and 0.2% (v/v) Triton X-100, pH 8.5.

Cytochrome c oxidase (CIV, EC 1.9.3.1) activity was measured at 550 nm to follow the oxidation of reduced cytochrome c (extinction coefficient ε_550_ = 29.5 ml cm^–1^ μmol^–1^) in a reaction medium containing 100 mM potassium phosphate, 0.05% (v/v) Tween 20 and 100 μM equine heart cytochrome c, pH 8.0. Just before analysis, cytochrome c was reduced with the addition of sodium dithionite without excess (4.5 mM final concentration). Samples were centrifuged at 3000 *g* (4°C) and the pellet was discarded prior to the analysis.

### Chemicals

Most chemicals were provided by Sigma Aldrich (Oakville, ON, Canada). EDTA, KCl, K_2_HPO_4_, and KH_2_PO_4_ were obtained from VWR (Ville Mont-Royal, QC, Canada), cytochrome c from equine heart was provided by Alfa Aesar (Tewksbury, MA, United States), and the protein standard was from Bio-Rad (Mississauga, ON, Canada).

### Calculations

Enzyme activities were normalized per protein unit. The bicinchoninic acid (BCA) method (Smith et al., 1985) was used to determine protein concentration. To compare the activity of enzymes from upstream of the ETS to the ETS, we used these enzyme ratios: PDHc/CI + CIII, PDHc/CIV, CPT/CI + CIII, CPT/CIV, HOAD/CI + CIII, HOAD/CIV, CPT/PDHc, HOAD/PDHc, PK/CI + CIII, PK/CIV, CI/CIV, CII/CIV, CI/CI + CIII, CI + CIII/CIV, CS/CI + CIII, and CS/CIV at the essay temperature closest to the species’ optimal temperature (5°C for the two species of notothen, 10°C for wolfish and cod, 15°C for Arctic char, 20°C for bass, and 25°C for tilapia and the carp). We expressed activities as ratios to characterize the organization of mitochondria instead of documenting only the content of each enzyme in different species. We also ran Pearson correlation analyses on enzyme activities (correlation between enzymes) and on the different ratios (ratios measured at close to optimal temperature correlated to the estimated optimal temperature). The objective was to have a picture of mitochondrial organization at different thermal conditions for the different fish species to test our hypothesis that fish adapted to cold environment have to compensate for the limitation at steps upstream of the ETS and therefore should express higher ratio of activities of enzymes responsible for entrance of electrons over the enzymes controlling the oxidative process.

### Statistics

All statistics were performed using the R platform (R Core Team, 2017). Significant differences between the activity ratios of the eight species were tested using one-way ANOVA. Tukey HSD tests were used to determine significant differences between groups. Normality and homogeneity of variances were analyzed using Shapiro-Wilk and Levene’s tests, respectively. When needed, a log_10_ transformation was used to respect the assumptions of the ANOVA. A statistical significance level of α = 0.05 was used for all tests. In all graphs, results are presented without transformations. We also performed a regression analysis to complement ANOVAs. The different ratios of activities measured close to optimal temperature were correlated with estimated optimal temperatures. Strong correlation would support the prediction of adjustments of the metabolic trait, expressed by a ratio, to environmental temperature.

## Results

**FIGURE 1:**
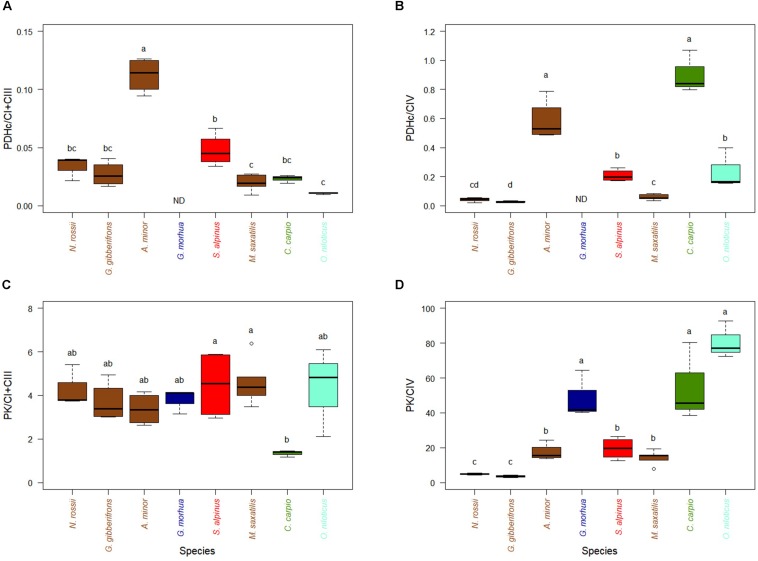
Activity of mitochondrial substrate entrance enzymes of pyruvate pathway (PDHc and PK) in hearts of cold-adapted fish (left), cold-temperate adapted fish, and warm-adapted fish (right). Activities were measured at temperature near their thermal optimum. **(A)** Pyruvate dehydrogenase complex (PDHc) expressed relative to CI + CIII activity. **(B)** PDHc expressed relative to CIV activity. **(C)** PK activity expressed relative to CI + CIII. **(D)** PK activity expressed relative to CIV. Values are represented as mean ± sem. Statistical differences are represented with letters (*p* < 0.05). ND: no data. *Notothenia rossii* (*n* = 3). *Gobionotothen gibberifrons* (*n* = 4). *Anarhichas minor* (*n* = 4). *Gadus morhua* (*n* = 3). *Salvelinus alpinus* (*n* = 4). *Morone saxatilis* (*n* = 5). *Cyprinus carpio* (*n* = 3). *Oreochromis niloticus* (*n* = 3). Brown boxes represent Perciformes, dark blue Gadiforme, red Salmoniforme, green Cypriniformes, and light blue is Cichliformes.

The capacity to feed the TCA cycle through PDHc or PK, when measured at a temperature close to the optimum of each species and compared to the activity of the ETS enzymes (CI + CIII or CIV), varied little among most of the species examined (Figures 1A–C), although the cold-temperate wolfish (*A. minor*) and the warm-adapted carp (*C. carpio*) showed higher relative levels of PDHc activity. Ratios of activities of PK over Complex IV, the last enzyme of glycolysis which feed mitochondria with pyruvate, were higher for species adapted to warmer temperature with the exception of *G. morhua* (Figure 1D). The correlation of PK/CIV with optimal temperature was high and significant (Supplementary Figure S2B, *R*^2^ = 0.56, *p* < 0.001).

**FIGURE 2:**
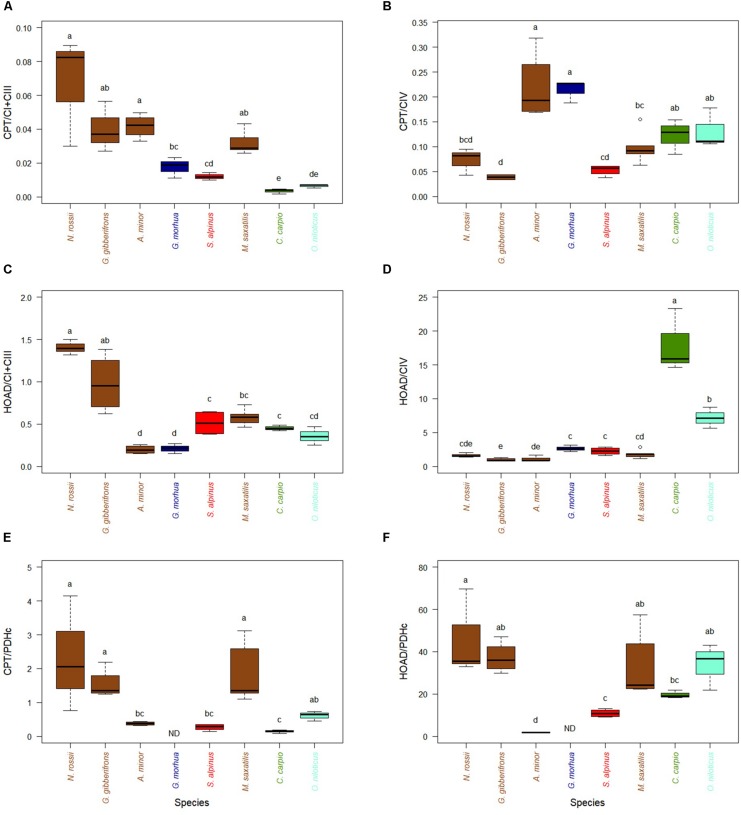
Activity of mitochondrial substrate entrance enzymes of pyruvate pathway (normalized with ETS enzyme activities (CI + CIII and CIV) or PDHc, in hearts of cold-adapted fish (left), cold-temperate adapted fish, and fish species adapted to warmer habitats (right). Enzyme activities were measured at temperature near their thermal optimum. **(A)** Carnitine palmitoyltransferase (CPT) expressed relative to CI + CIII. **(B)** CPT expressed relative to CIV. **(C)** 3-Hydroxyacyl-CoA dehydrogenase (HOAD) expressed relative to CI + CIII. **(D)** 3-HOAD expressed relative to CIV. **(E)** CPT expressed relative to pyruvate dehydrogenase complex (PDHc). **(F)** 3-HOAD expressed relative to PDHc. Values are represented as mean ± SEM. Statistical differences are represented with letters (*p* < 0.05). *Notothenia rossii* (*n* = 3). *Gobionotothen gibberifrons* (*n* = 4). *Anarhichas minor* (*n* = 4). *Gadus morhua* (*n* = 3). *Salvelinus alpinus* (*n* = 4). *Morone saxatilis* (*n* = 5). *Cyprinus carpio* (*n* = 3). *Oreochromis niloticus* (*n* = 3). Brown boxes represent Perciformes, dark blue Gadiforme, red Salmoniforme, green Cypriniformes, and light blue is Cichliformes.

The capacity to feed TCA by fatty acid oxidation, appears higher in cold-adapted fish since the activities of CPT, relative to CI + CIII are greater for the three species with lowest optimal temperatures when compared to four of the five species adapted to higher temperatures (Figure 2A). This difference was lost when CPT was normalized to CIV activity (Figure 2B). Two cold-temperate species, *A. minor* and *G. morhua*, had the highest relative CPT/CIV activities. The activity of HOAD, a key enzyme of the β-oxidation pathway, relative to CI + CIII, partly mimicked the differences observed for CPT (Figure 2C). However, this trend disappeared again when HOAD was normalized to CIV activity, with two warm-adapted species (*C. carpio* and *O. niloticus*) expressing higher relative activities (Figure 2D). Interestingly, the relative activities of either CPT or HOAD over PDHc did not show any specific trend with temperature (Figures 2E,F). Capacities to oxidize fatty acids, compared to pyruvate oxidation, were, however, greater in two cold-adapted species (*N. rossii* and *G*. *gobionotothens*) and a temperate species (*M. saxatilis*). The correlations of key enzymes of metabolites entrance in oxidative phosphorylation, with CI + CIII or CIV (Supplementary Figure S5) show the same relations. They reveal a strong correlation of HOAD activity with CI + CIII (Supplementary Figure S5D).

**FIGURE 3:**
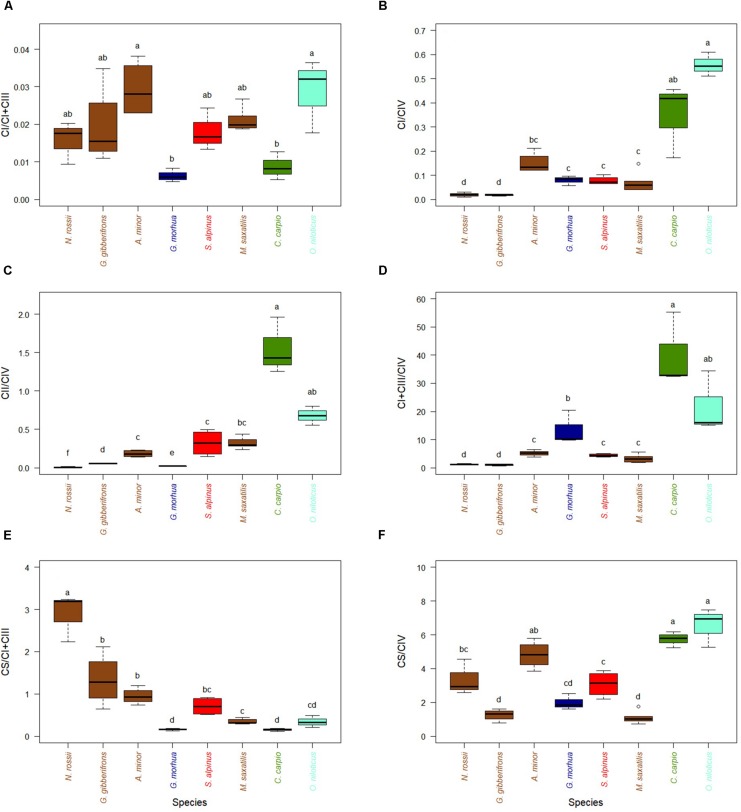
Activity ratios of CS and ETS enzymes in hearts of cold-adapted fish (left), cold-temperate adapted fish and fish species adapted to warmer habitats (right). Enzymes activities were measured at temperature near their thermal optimum. **(A)** CI activity expressed relative to CI + CIII. **(B)** CI activity expressed relative to CIV. **(C)** CII activity expressed relative to CIV. **(D)** CI + CIII activity expressed relative to CIV. **(E)** CS activity expressed relative to CI + CIII. **(F)** CS activity expressed relative to CIV. Values are represented as mean ± SEM. Statistical differences are represented with letters (*p* < 0.05). *Notothenia rossii* (*n* = 3). *Gobionotothen gibberifrons* (*n* = 4). *Anarhichas minor* (*n* = 4). *Gadus morhua* (*n* = 3). *Salvelinus alpinus* (*n* = 4). *Morone saxatilis* (*n* = 5). *Cyprinus carpio* (*n* = 3). *Oreochromis niloticus* (*n* = 3). Brown boxes represent Perciformes, dark blue Gadiforme, red Salmoniforme, green Cypriniformes, and light blue is Cichliformes.

With respect to the ETS, activities of CI relative to CIV were higher for the two warm-adapted species and lower for the cold-adapted species, and this trend was repeated for CII (Figures 3B,C), for example *C. carpio* showed higher activity than *O. niloticus*. When normalized to CI + CIII, activities of CI did not display any trend with thermal optimum of the different species (Figure 3A). Relative activities of CI + CIII normalized to CIV were greater for warm-adapted species (*C. carpio* and *O. niloticus*) than for the other species at their optimal temperatures (Figure 3D). Interestingly, when normalizing activities with either CI + CIII or CIV, very different and often opposite trends with estimated optimal temperatures were observed. CPT, HOAD, and CS all decreased with increases in optimal temperature when normalized per CI + CIII while HOAD, PK, CS as well as CI and CII increased when normalized per CIV (see Supplementary Figures S1–S4).

Activities of CS, a TCA cycle enzyme, tended to decrease with optimal species temperature when compared to activities of CI + CIII (Figure 3E). This is also observed when CS/CI + CIII ratios are correlated with optimal temperature (Supplementary Figure S4B, *R*^2^ = 0.48, *p* < 0.001). For CS/CIV ratio (Figure 3F) the correlation with optimal temperature was significantly reversed but the relationship was not as strong (Supplementary Figure S4A, *R*^2^ = 0.26, *p* < 0.003). When correlating the different enzymes measured at optimal temperatures to identify those for which activities co-vary (Supplementary Figures S5, S6), the only correlation we identified was HOAD with CI + CIII (Supplementary Figure S5D, *R*^2^ = 0.82; *p* < 0.001) and CII with CIV (Supplementary Figure S6C, *R*^2^ = 0.62; *p* < 0.001). Enzyme activities (expressed per mg of proteins) for each species and measured at different temperatures are presented in Supplementary Figures S7–S10.

## Discussion

In this report, we characterize the cardiac mitochondrial pathways of eight ray-finned fish species that inhabit thermal niches spanning most of the range occupied by the Actinopterygii. Our goal was to identify potential evolutionary adjustments of mitochondrial metabolism in fish taxa across a wide range of thermal habitats. Knowing key characters of mitochondria associated to specific thermal niche should help to point out steps needed to adapt to changes in habitat temperatures and therefore refine further studies on adaptability of ectotherms to temperature.

Prior work on the thermal sensitivity of mitochondrial metabolism in fish have often focused on enzymes of OXPHOS, the PDHc and some enzymes of TCA cycle such as CS, but have largely neglected fatty acid oxidation pathways (Takeuchi et al., 2009; Lemieux et al., 2010a, b; Blier et al., 2014; but see Ekström et al., 2017). Here, we have investigated enzymes that control substrate entrance, including lipids and carbohydrates, into the TCA cycle, as well as enzyme complexes of the electron transport chain. PDHc, the gatekeeper of the glucose oxidation pathway, showed no evidence of compensation, relative to ETS enzyme activities, in cold-adapted fish species, compared to temperate or warm adapted species, refuting our prediction based on prior work (Takeuchi et al., 2009; Lemieux et al., 2010a, b). Rather, the activity of CPT, a gatekeeper of the fatty acid oxidation pathway, when normalized with respect to the ETS (CI + III), revealed a strong compensation of fatty acid oxidation in heart mitochondria of cold-water fish species. Two of the three species adapted to the coldest environment showed ratios more than two times higher than those of four of the five species adapted to higher temperature. This is even more evident when the ratios are correlated to the estimated optimal temperatures. Similarly, HOAD activity appeared to be adjusted, with some variation, to maintain fatty acid oxidation capacity at low temperature. The correlation of HOAD with CI + CIII activities among species might suggest a coevolution in the regulation of the expression of these enzymes leading to coordination of fatty acid oxidation capacity with ETS. The ratio obtained for either CPT or HOAD over CI + III were, however, calculated with activities measured at 5°C in both cold-adapted notothenioid fish due to the technical difficulties to measure at 0°C even though these fish are adapted to temperature close to or below 0°C. A temperature of 5°C is significantly higher than “optimal” temperature and these ratios may represent heat shock conditions. To examine this possibility as well as the physiological significance of the ratios obtained from assays at 5°C, we estimated the activities of enzymes at 0°C by extrapolating from regression of activities at 5, 10, and 15°C. From these extrapolations the CPT/CI + III ratio is lower at 0°C in *N. rossii* than at 5°C (0.03 instead of 0.06) with no significant difference between both temperatures. In *G. gibberifrons*, the ratio from extrapolated activities at 0°C is, however, almost three times higher than at 5°C (0.11 instead of 0.04, with 0.11 being outside the range of standard deviation of the ratio at 5°C). Ratios calculated for 0°C are still higher than the ratios of four of the five fish species adapted at higher temperatures. For the ratios of HOAD over CI + III., extrapolations at 0°C show a higher ratio only for *G. gibberifrons* (1.3 instead of 0.9 but within the range of standard deviation of the ratio at 5°C). These observations suggest that temperature sensitivity of lipid oxidation, which provides a high proportion of the energy requirements of fish heart, could be compensated at key steps (CPT and HOAD) likely to maintain mitochondrial capacity at low temperature. All the fish were collected or acclimated at temperatures close to the estimated optimal temperatures. We suspect that these ratios approximate mitochondrial characters allowing optimal capacity to oxidize fatty acids in the range of temperature encountered during their life cycle. Comparison of the activity of PK, the final glycolytic enzyme, to complexes I + III suggests that the relative capacity to provide pyruvate to mitochondria is conserved among the eight species at their respective optimal temperatures. However, warm-living fish have PK/CIV ratios that are much larger than those of cold-adapted species.

The relationship of the ratios of gatekeeper enzymes over ETS enzymes with temperature is, however, dependent on the complex used to normalize the activity (denominator of the ratio). As mentioned CPT, HOAD, and CS decrease with increases in optimal temperature when normalized per CI + CIII while HOAD, PK, CS as well as CI and CII increase when normalized per CIV. This is partly explained by the relation of CI + CIII with CIV at different temperatures of adaptation. The ratio of CI + CIII over CIV increases with increases in optimal temperatures (Supplementary Figure S6B). This clearly shows that the organization of the ETS in fish heart mitochondria is variable and appears to be associated to environmental temperature. The predicted pattern of organization (higher gatekeeper enzymes over oxidative capacity) is revealed only when their activities are normalized by CI + CIII.

In mouse heart, Lemieux et al. (2017) measured a weak control of oxidative phosphorylation by Cytochrome c Oxidase at physiological temperature while they showed Lemieux et al. (2008) that the thermal sensitivity of cytochrome c oxidase cannot drive the thermal sensitivity of mitochondrial respiration, ruling out significant control of oxidative phosphorylation at this level. An excess of cytochrome c oxidase capacity relative to OXPHOS of at least 1.5-fold has been reported for the heart mitochondria of three fish species (*Bellapiscis medius*, *Forsterygion varium*, and *F. malcolmi*) at three temperatures [15, 25, and 30°C; Hilton et al. (2010)]. Much higher activities of Cytochrome c oxidase than the whole Electron Transfer System have also been reported in the heart of three species of wrasses (*Notolabrus celidotus*, *Notolabrus fucicola*, and *Thalassoma lunare*), Iftikar et al., 2014. This excess of Cytochrome c Oxidase may suggest low control by CIV over maximal respiration (but see Blier and Lemieux, 2001). If CIV insures low level of control over the maxima catalytic capacity of mitochondria, the ratios expressed per CI + III activity might be more physiologically significant and the trend observed among species could reveal a higher increased capacity to provide electrons to CI and CII through fatty acid oxidation. The increase in the ratio of CI + III/CIV at higher temperatures is, however, intriguing. One hypothesis is that ETS over CIV ratio could evolve to insure proper reduction state and associated catalytic capacity and regulatory properties.

Compensation of mitochondrial energy production in cold-living fish can occur at the organellar level via increases in mitochondrial volume density (Strobel et al., 2013), greater inner membrane surface density (St.-Pierre et al., 1998), and modifications in membrane composition (Kraffe et al., 2007; Grim et al., 2010). Mitochondrial content is often evaluated using CS activity, and cristae surface area can be estimated from CIV activity (Larsen et al., 2012). The trend observed in our CS/CI + CIII ratio data suggest higher volume/surface ratio for cold-adapted species, consistent with higher mitochondrial content, and an increase in mitochondria surface density in warm-adapted species. However, based on the CS/CIV ratios, the species studied are likely to have similar mitochondrial volume/surface ratios, with the exception of the two warm-adapted species. We suggest that thermal adaptation of mitochondrial metabolism in fish most likely results from compensation at the level of enzymatic properties, quantities and metabolic organization rather than organellar quantity or volume.

Homeoviscous adaptation through changes in the lipid composition of mitochondrial membranes can play an important role in acclimation and adaptation of energy metabolism to cold and warm temperatures (Guderley and St-Pierre, 2002; Kraffe et al., 2007; Pörtner et al., 2007; Grim et al., 2010; Hofmann and Todgham, 2010). Such remodeling can have big impacts on the activities of enzymes that are embedded in the mitochondrial membranes, including CPT and the four respiratory complexes. However, our data cannot address whether the activities of these enzymes from the species that we have examined are modulated through temperature-dependent adjustments of membrane composition and properties.

In this study, we have characterized heart mitochondrial phenotypes from eight fish species adapted to different thermal habitats by comparing the activities of enzymes from different steps of mitochondrial respiration and electron transport. We suggest that natural selection driven by habitat temperature has shaped mitochondrial function and regulation (Blier et al., 2014; Lemieux et al., 2017) such that distinct mitochondrial pathways allow cold- and warm-living fish to optimize aerobic capacity and regulation. Our results demonstrate important variability of organization in the heart mitochondrial pathway among fish species of various thermal habitats. Among the characters, two appear related to optimal temperature; ratios of CPT/CI + CIII and ratios of HOAD/CI + CIII. These increases in activities of fatty acid oxidation enzymes in cold adapted fish are complemented by increase in a key enzyme of TCA (CS). It is unlikely that these relationships are induced by phylogenetic relationship since fish from four basal orders (Gadiformes, Salmoniformes, Cyprinoformes, and Cichliformes) cover an optimal temperature range from 9 to 30°C while the four other species are all from the Perciforme order and cover a temperature range from 0 to 18°C, with the Cichliformes (*O. niloticus*; 30°C) being closer to Perciformes than to others. Furthermore, the optimal temperatures in the perciformes fish from the present study are independent of the phylogenetic distance (see the phylogeny of Hughes et al., 2018). The strong correlation observed between CPT/CI + CIII or HOAD/CI + CIII and optimal temperature could therefore likely be associated to adaptation to the temperature regime. These results are in line with the demonstration of higher ability to oxidize fatty acids in the muscle of two temperate fish species (*M. saxatilis* and *O. mykiss*) following acclimation to low temperature (Rodnick and Sidell, 1994; St.-Pierre et al., 1998). Before concluding that these ratios represent clear improvement of the ability to oxidize fatty acids in cold-adapted species through increments of gatekeeper steps, we need to know which character sets the oxidative capacity of mitochondria (CI + CIII or CIV). One way to address this question would be to estimate the control strength of different steps of mitochondrial pathways and to compare the ability to oxidize fatty acids at different temperatures in different species from a wide range of thermal habitats.

The changing thermal environments that will result from climate change might therefore affect mitochondrial functions differently according to the thermal niche of the species. For example, a fish species adapted to cold environment may therefore be more affected by the thermal sensitivity of gate keepers of fatty acid oxidation (CPT) while warm adapted species could be more impaired by the impact of temperature on CIV. A corollary of this is that following rapid temperature changes, populations might face an excess of these key enzymes potentially resulting in an increase in reduction status of ETS and thus a burst of ROS production (see Christen et al., 2018). This study clearly reveals the urgency of further studies scrutinizing the extent to which selection could remodel mitochondrial organization rather than only accommodating mitochondrial content.

## Ethics Statement

This study was carried out in accordance with the principles of the Basel Declaration and recommendations of Canadian Council of Animal Care. The protocol was approved by the Comité de Protection des Animaux de l’UQAR.

## Author Contributions

FH-M carried out the experiments and statistical analyses, and drafted the manuscript. PB designed the study, obtained the funding, supervised the analysis, and revised the manuscript. VD supervised FH-M during biochemical analysis. NL and HD conducted the field research program that obtained the Antarctic fish samples and revised the manuscript. NL sampled Arctic fish and provided the tilapia. FD and CN participated in the analysis and revised the manuscript. HD helped to get access to the biological material and revised the manuscript.

## Conflict of Interest

The authors declare that the research was conducted in the absence of any commercial or financial relationships that could be construed as a potential conflict of interest.
