# Supplementary material for: From Africa to Antarctica: Exploring the Metabolism of Fish Heart Mitochondria Across a Wide Thermal Range
Source: Front Physiol. 2019 Oct 4;10:1220. doi: 10.3389/fphys.2019.01220 (PMC6788138; doi:10.3389/fphys.2019.01220)
Supplement: Supplementary file 1 [file Image_1.pdf]

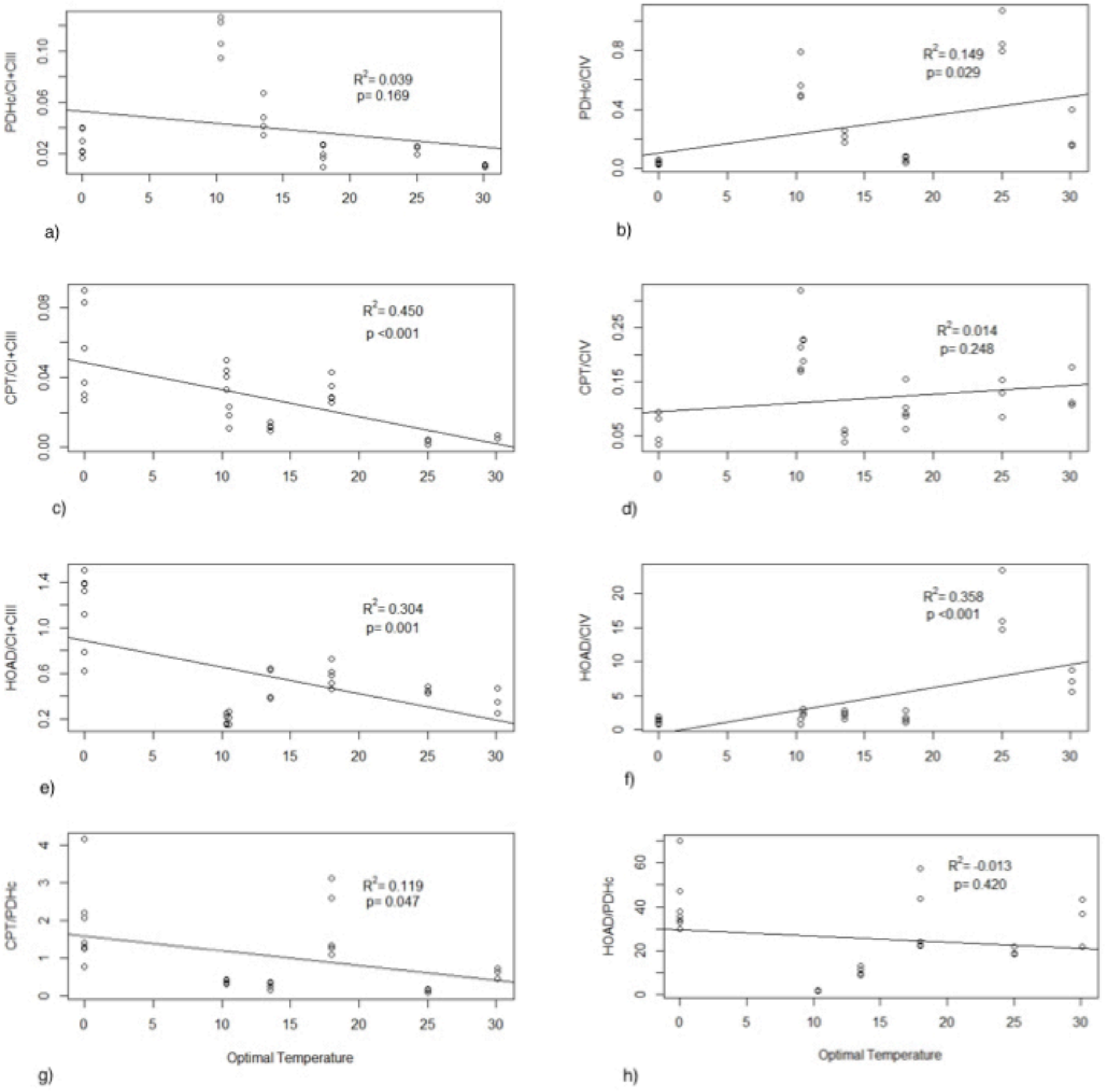

Figure S1. Correlation of activities of key enzymes of substrates entrances into mitochondrial pathway, with the estimated optimal temperature (°C) of each species, normalized by ETS complexes activities, [a) PDHc/CI+CIII, b) PDHc/CIV, c) CPT/CI+CIII, d) CPT/CIV, e) HOAD/CI+CIII, f) HOAD/CIV] or when normalized by the activity of another key enzyme of substrate entrance [g) CPT/PDHc, h) HOAD/PDHc].
