# Supplementary material for: From Africa to Antarctica: Exploring the Metabolism of Fish Heart Mitochondria Across a Wide Thermal Range
Source: Front Physiol. 2019 Oct 4;10:1220. doi: 10.3389/fphys.2019.01220 (PMC6788138; doi:10.3389/fphys.2019.01220)
Supplement: Supplementary file 2 [file Image_2.pdf]

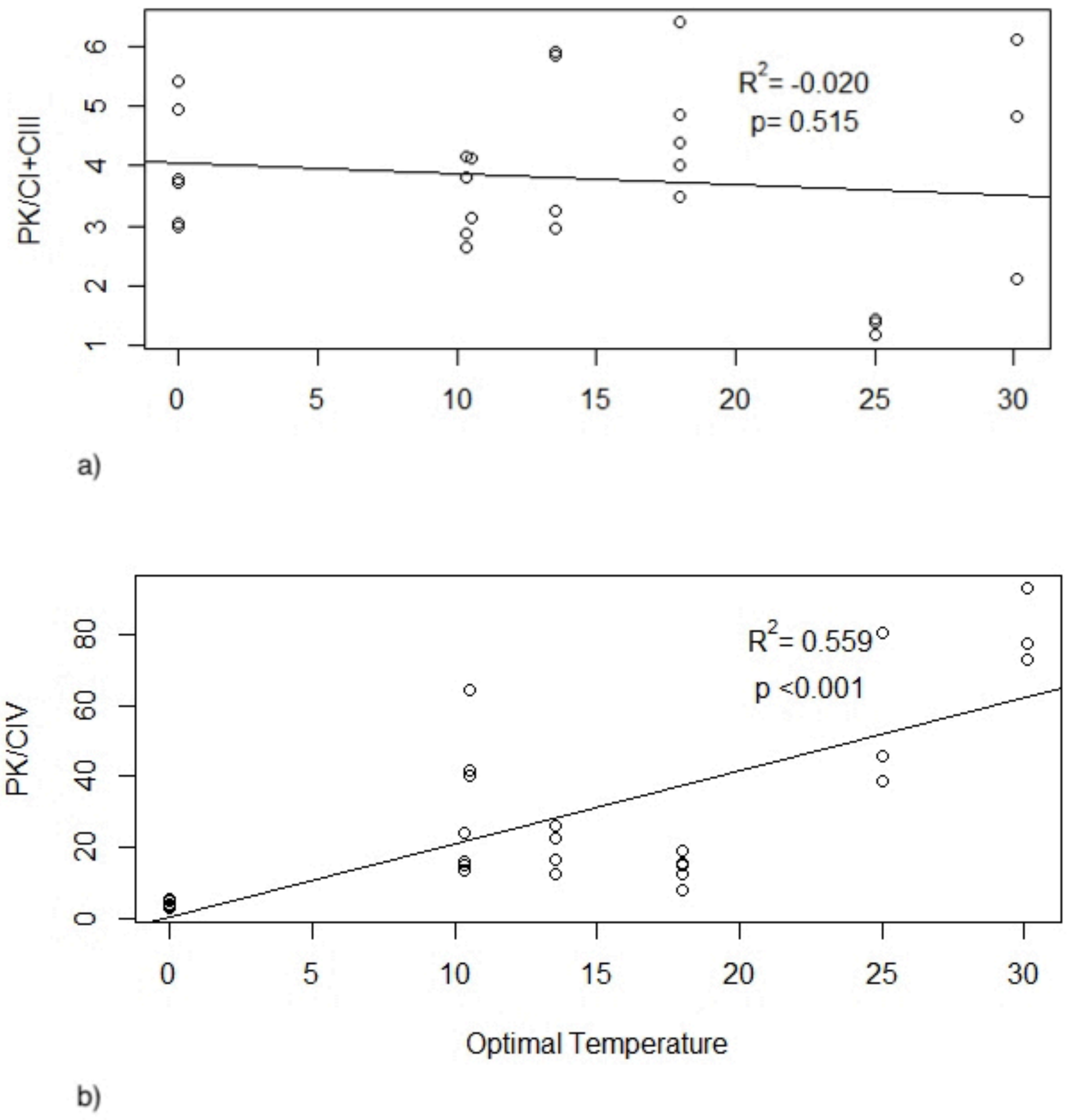

Figure S2. Correlation of PK activity, when normalized with either CI+CIII (a) or CIV (b), with estimated optimal temperature of species (°C).
