# Supplementary material for: From Africa to Antarctica: Exploring the Metabolism of Fish Heart Mitochondria Across a Wide Thermal Range
Source: Front Physiol. 2019 Oct 4;10:1220. doi: 10.3389/fphys.2019.01220 (PMC6788138; doi:10.3389/fphys.2019.01220)
Supplement: Supplementary file 3 [file Image_3.pdf]

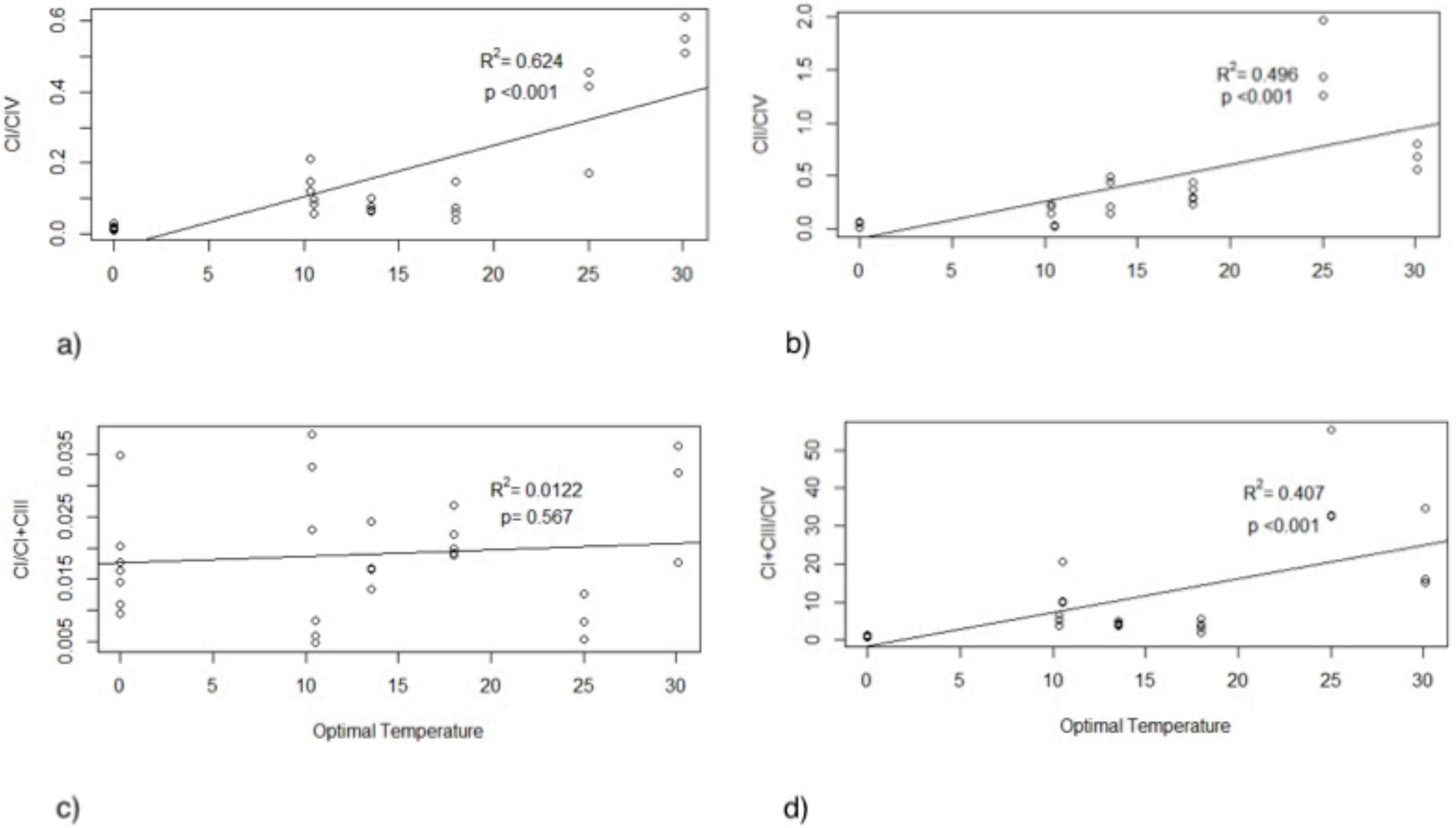

Figures S3. Correlations of relative activities of enzymes of the Electron Transport System, normalized by either CI+CIII or CIV activities, with estimated optimal temperatures of species (°C). a) CI/CIV, b) CI/CIV, c) CI/CI+CIII, d) CI+CIII/CIV.
