# Supplementary material for: From Africa to Antarctica: Exploring the Metabolism of Fish Heart Mitochondria Across a Wide Thermal Range
Source: Front Physiol. 2019 Oct 4;10:1220. doi: 10.3389/fphys.2019.01220 (PMC6788138; doi:10.3389/fphys.2019.01220)
Supplement: Supplementary file 4 [file Image_4.pdf]

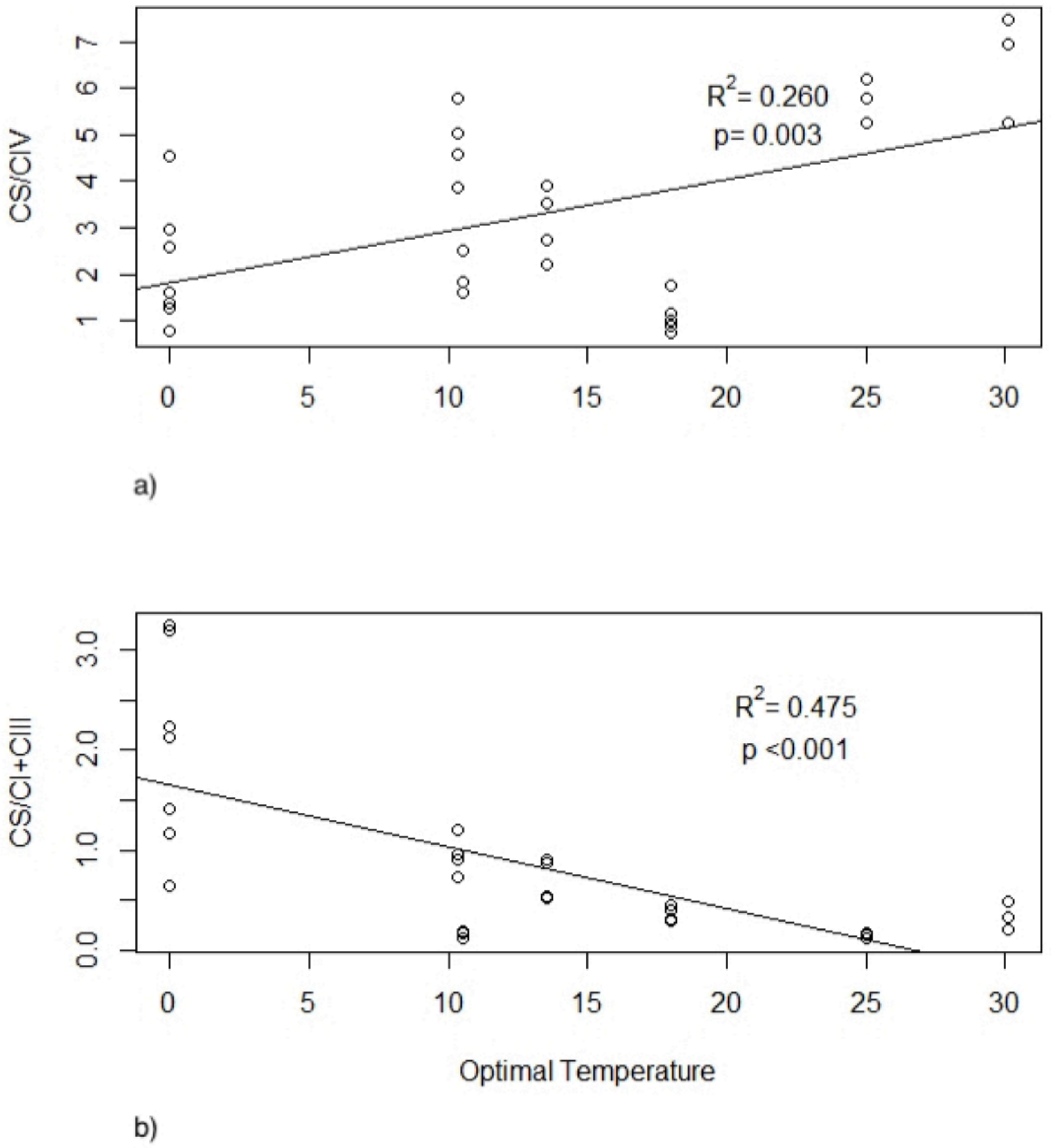

Figure S4. Correlations of CS activities, normalized by either CIV or CI+CIII, with the estimated optimal temperatures of different species (°C). a) CS/CIV, b) CS/CI+CIII
