# Supplementary material for: From Africa to Antarctica: Exploring the Metabolism of Fish Heart Mitochondria Across a Wide Thermal Range
Source: Front Physiol. 2019 Oct 4;10:1220. doi: 10.3389/fphys.2019.01220 (PMC6788138; doi:10.3389/fphys.2019.01220)
Supplement: Supplementary file 5 [file Image_5.pdf]

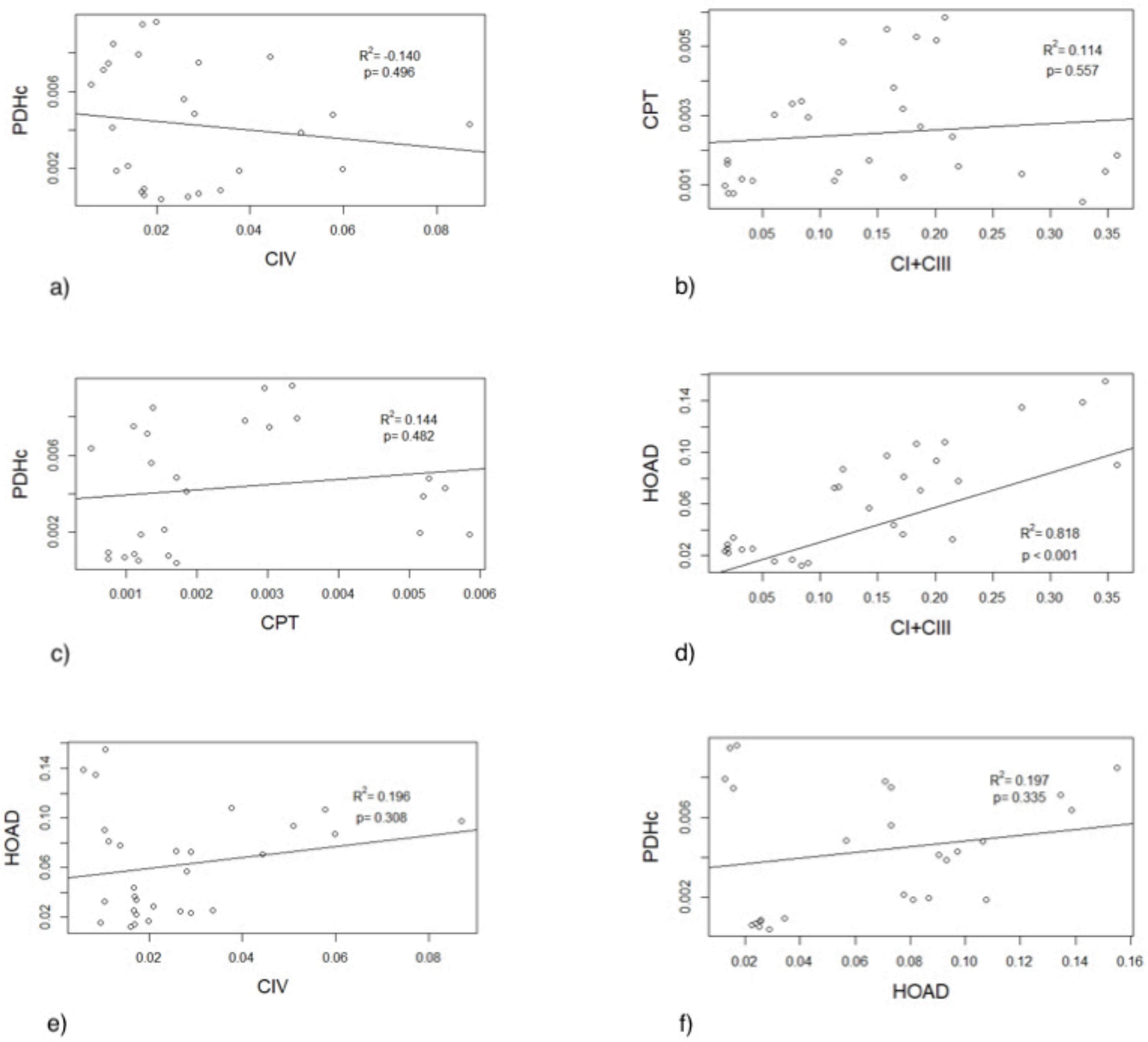

Figures S5. Correlation in activities among different enzymes of mitochondrial oxidative pathway. a) PDHc correlated with CIV, b) CPT correlated with CI+CIII, c) PDHc correlated with CPT, d) HOAD correlated with CI+CIII, e) HOAD correlated with CIV, f) PDHc correlated with HOAD.
