# Supplementary material for: From Africa to Antarctica: Exploring the Metabolism of Fish Heart Mitochondria Across a Wide Thermal Range
Source: Front Physiol. 2019 Oct 4;10:1220. doi: 10.3389/fphys.2019.01220 (PMC6788138; doi:10.3389/fphys.2019.01220)
Supplement: Supplementary file 6 [file Image_6.pdf]

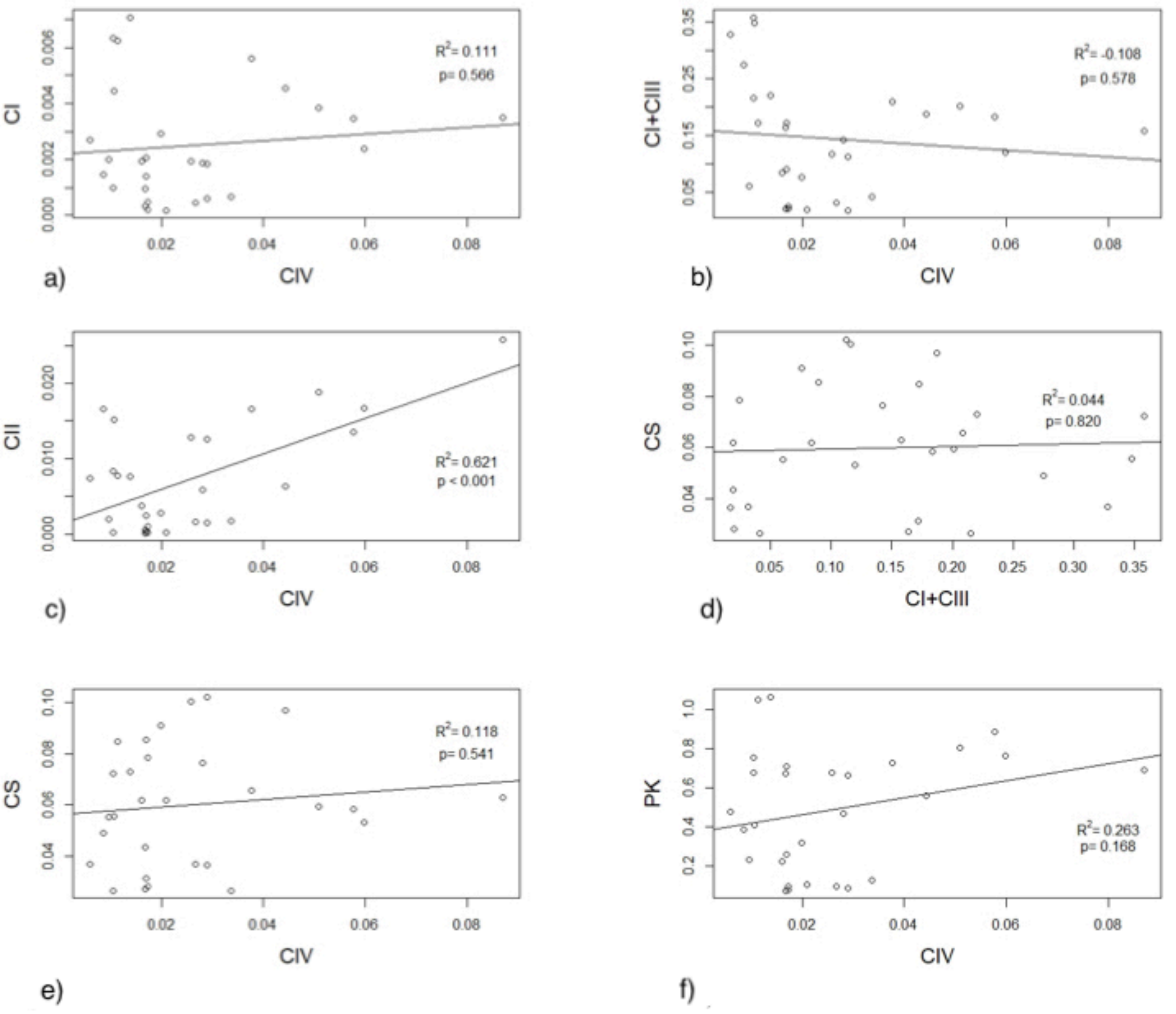

Figures S6. Correlation in activities among different enzymes of mitochondrial oxidative pathway. a) CI correlated with CIV, b) CI+CIII correlated with CIV, c) CII correlated with CIV, d) CS correlated with CI+CIII, e) CS correlated with CIV f) PK correlated with CIV.
