# Supplementary material for: From Africa to Antarctica: Exploring the Metabolism of Fish Heart Mitochondria Across a Wide Thermal Range
Source: Front Physiol. 2019 Oct 4;10:1220. doi: 10.3389/fphys.2019.01220 (PMC6788138; doi:10.3389/fphys.2019.01220)
Supplement: Supplementary file 7 [file Image_7.pdf]

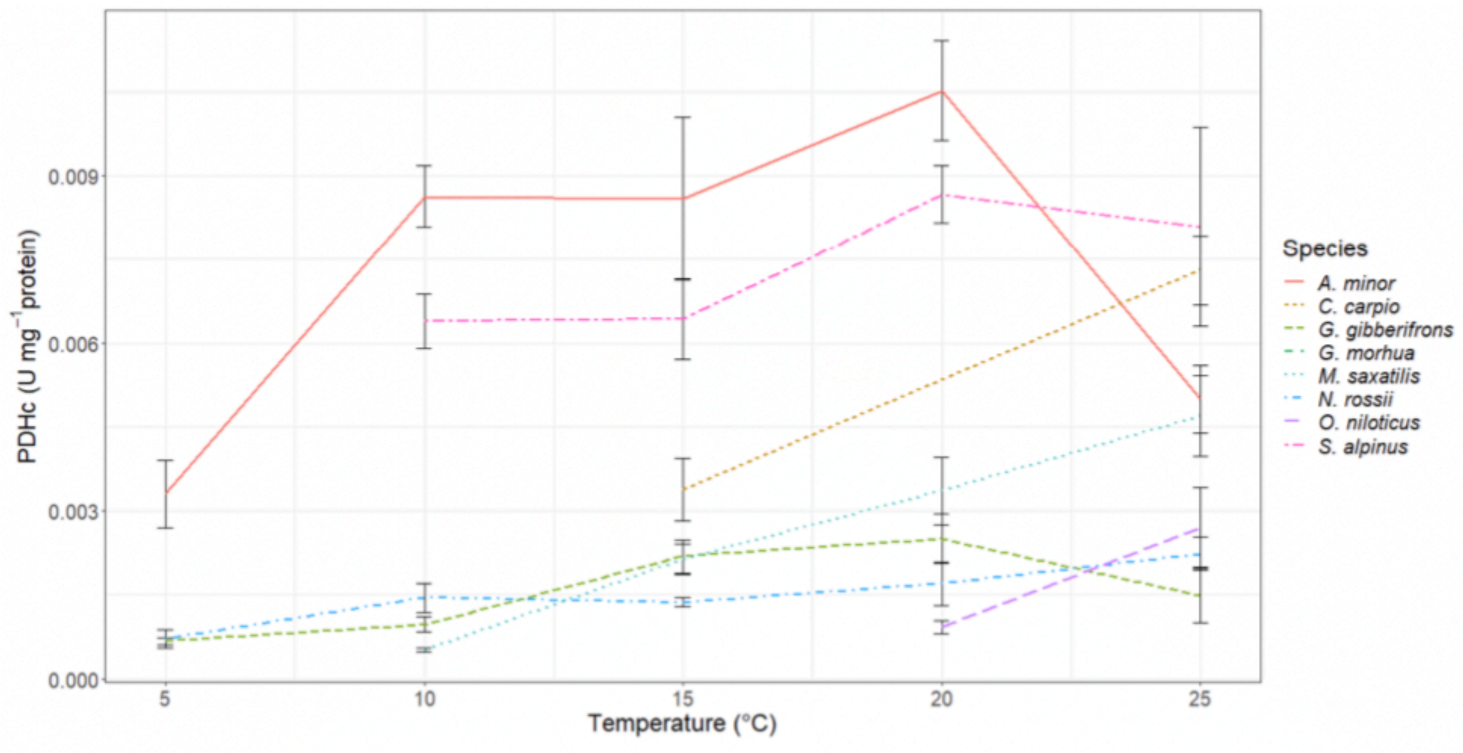

a)

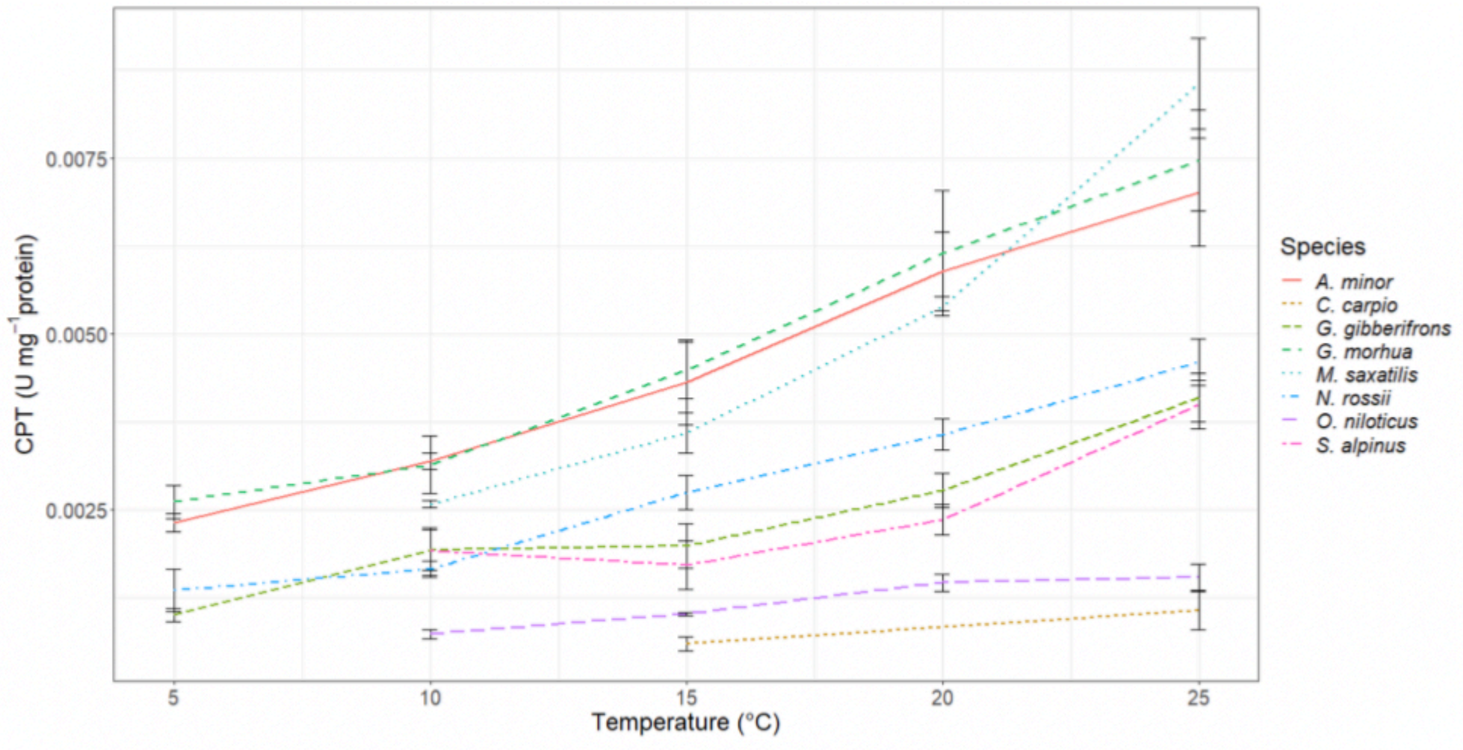

b)

Figure S7. Activity of two key enzymes of substrates entrance (carbohydrates and fatty acids) in mitochondrial oxidative pathway, in height species of fish measured at five different temperatures. a) PDHc, b) CPT.
