# Supplementary material for: From Africa to Antarctica: Exploring the Metabolism of Fish Heart Mitochondria Across a Wide Thermal Range
Source: Front Physiol. 2019 Oct 4;10:1220. doi: 10.3389/fphys.2019.01220 (PMC6788138; doi:10.3389/fphys.2019.01220)
Supplement: Supplementary file 8 [file Image_8.pdf]

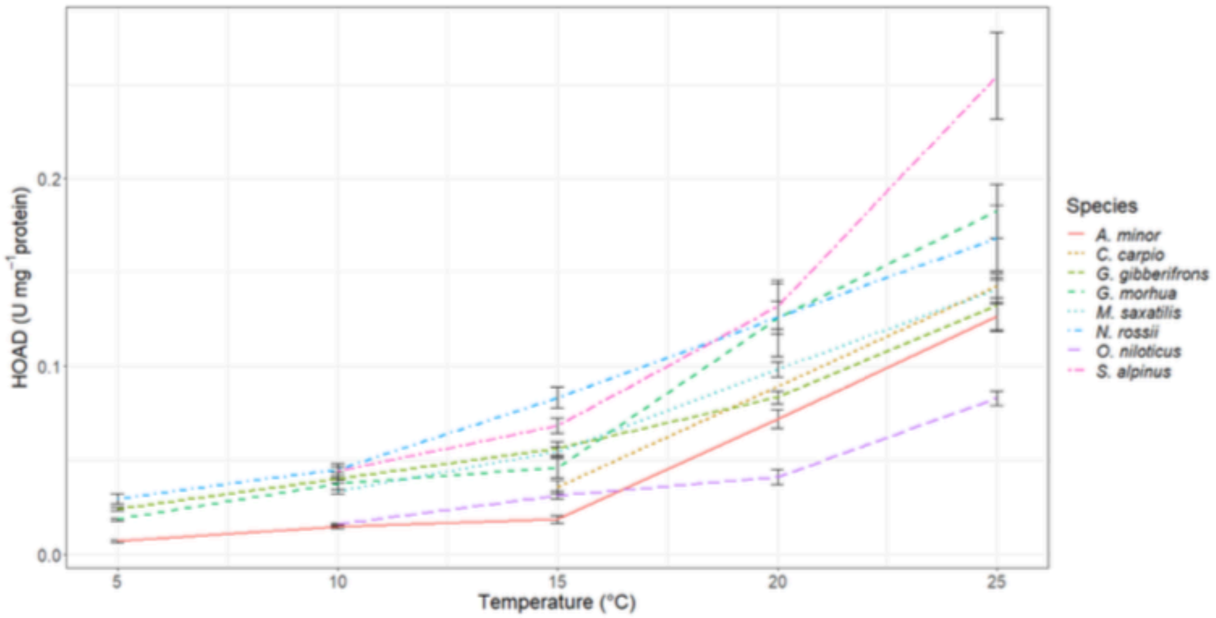

a)

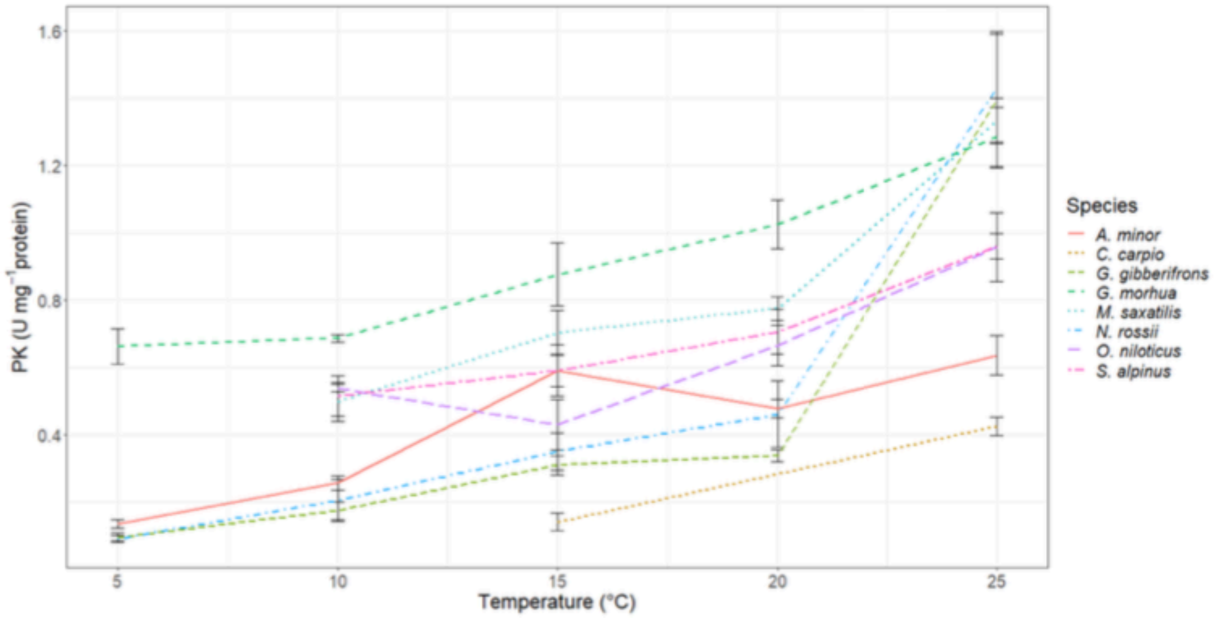

b)

Figures S8. Activities of HOAD (a) and PK (b) measured at different temperatures in height fish species.
