# Supplementary material for: From Africa to Antarctica: Exploring the Metabolism of Fish Heart Mitochondria Across a Wide Thermal Range
Source: Front Physiol. 2019 Oct 4;10:1220. doi: 10.3389/fphys.2019.01220 (PMC6788138; doi:10.3389/fphys.2019.01220)
Supplement: Supplementary file 9 [file Image_9.pdf]

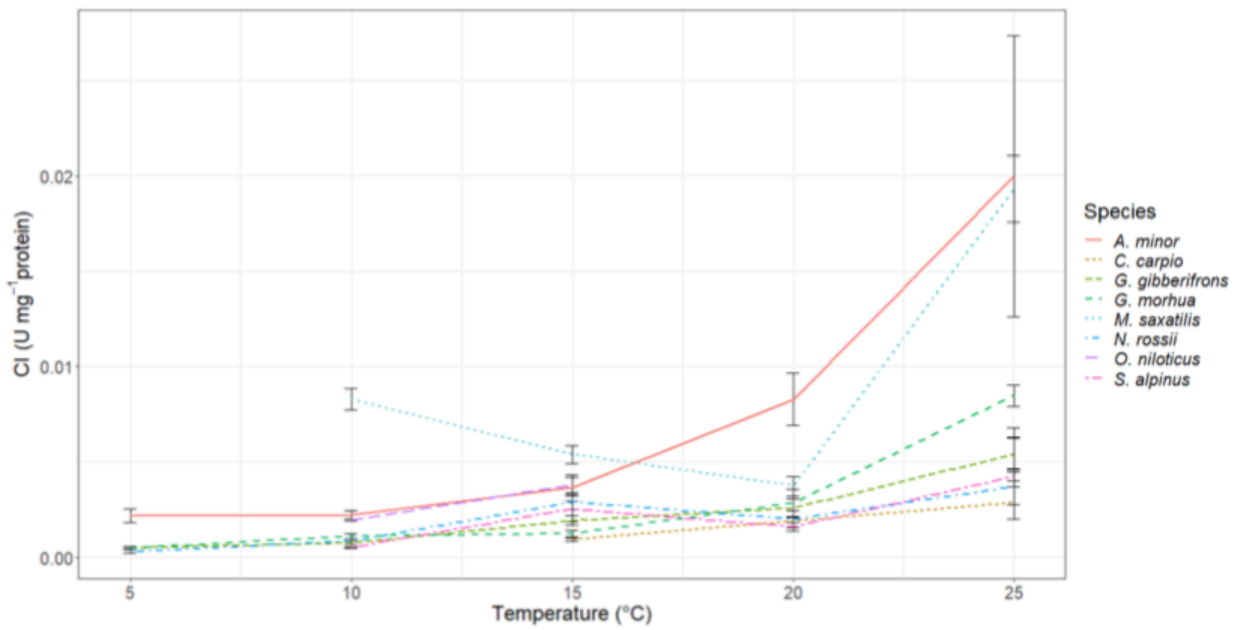

a)

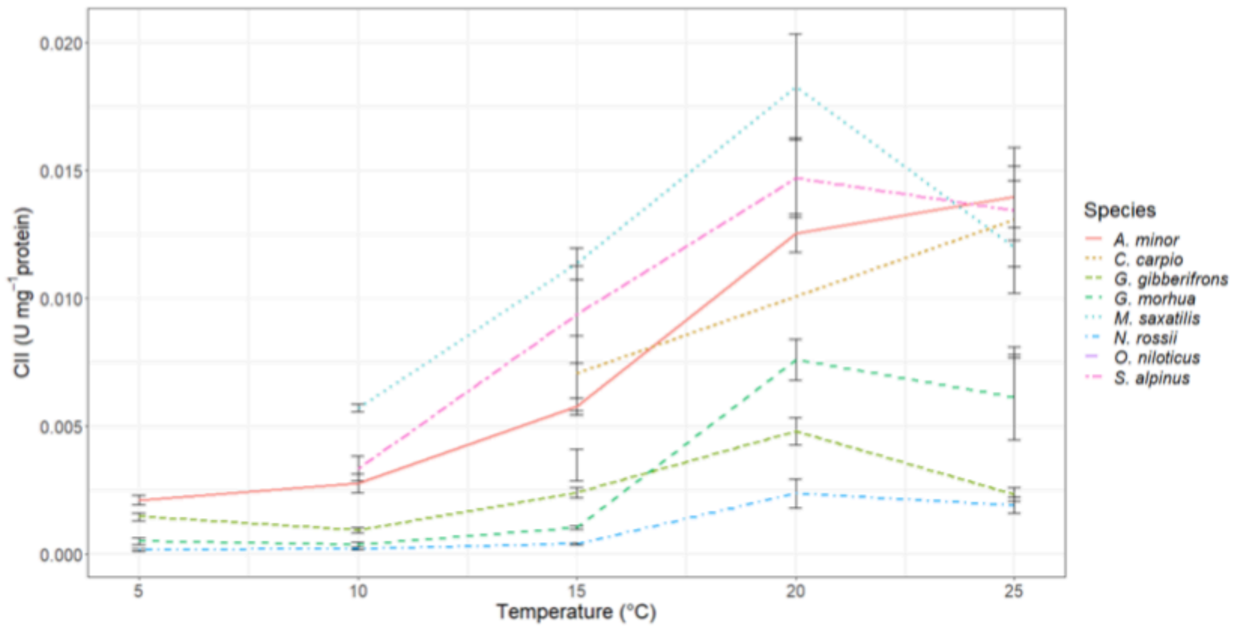

b)

Figures S9. Activities of two key enzymes of electron entrance in the Electron transport System, CI (a) and CII (b) measured in the heart of height species of fish at five different temperatures.
