# Supplementary material for: From Africa to Antarctica: Exploring the Metabolism of Fish Heart Mitochondria Across a Wide Thermal Range
Source: Front Physiol. 2019 Oct 4;10:1220. doi: 10.3389/fphys.2019.01220 (PMC6788138; doi:10.3389/fphys.2019.01220)
Supplement: Supplementary file 10 [file Image_10.pdf]

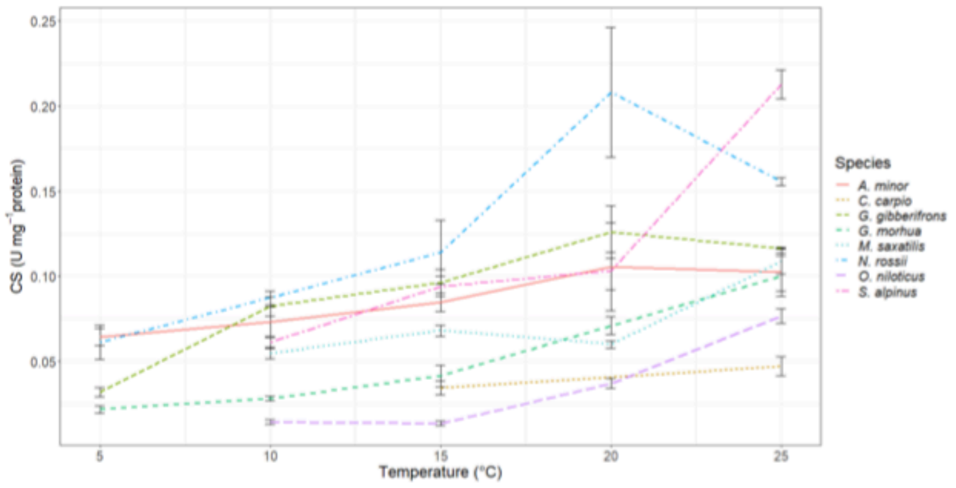

a)

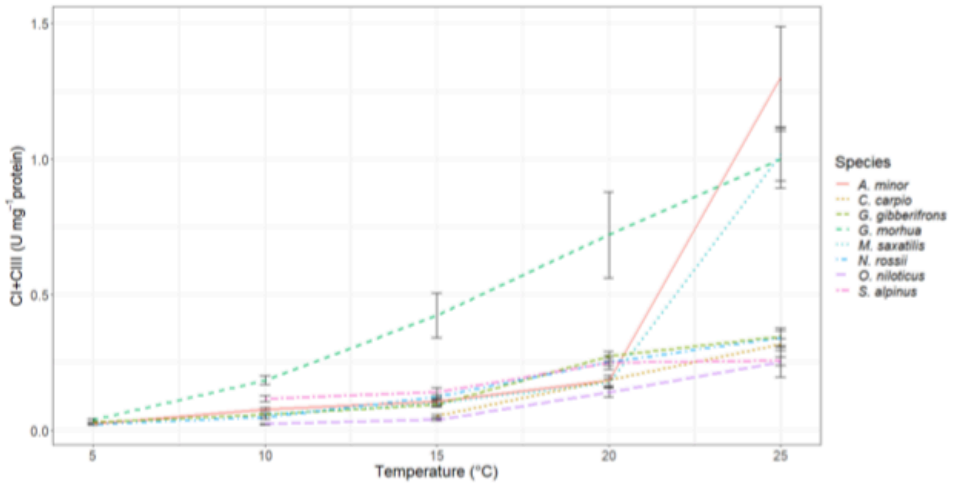

b)

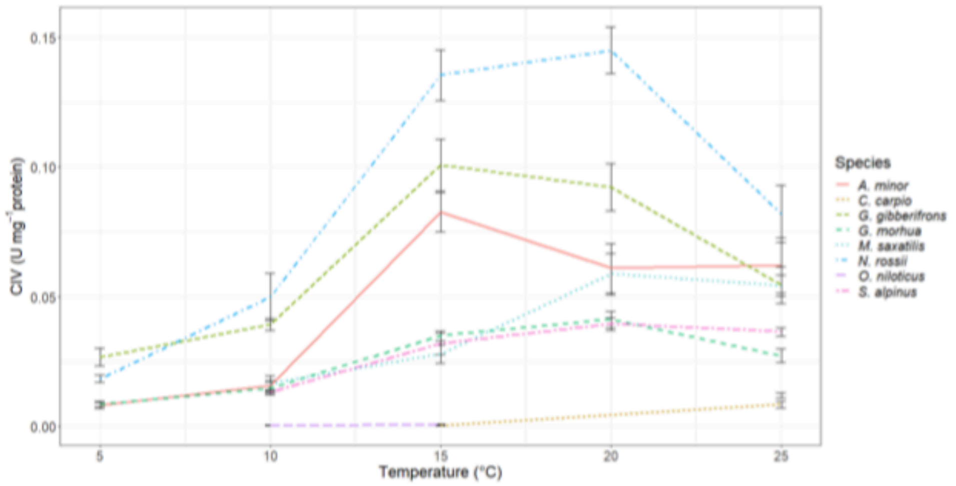

c)

Figures S10. Activities of one enzyme of the Tricarboxylic Acid Cycle [a) CS], and two enzyme complexes of the ETS [b) CI+CIII, c) CIV], measured in the heart of height fish species and at five temperatures.
